# Discussions of the Mississippi Valley Dental Association

**Published:** 1876-07

**Authors:** F. W. Sage


### DISCUSSIONS OF THE MISSISSIPPI VALLEY DENTAL ASSOCIATION.

REPORTED BY F. W. SAGE, D. D. S.

WEDNESDAY MORNING, MARCH 1, 1876.

The sixth subject on the programme, viz. "Mechanical Dentistry, including irregularities of the teeth," was taken up for consideration.

Dr. Craven reported a case of the loss of the palate. The patient is a lady sixty years old. She has lost the entire roof of mouth, hard and soft palate from syphilis. There is an edge formed around the margin of the opening by the alveolar process; the inferior turbinated bone is visible. The lower teeth are hardly reliable as a point of attachment for old fashioned springs, to be used in supporting the appliance; the lower teeth will probably all be lost by absorption in a few months; the vellum can be held in position very easily, I think; there are no teeth in the upper jaw.

Dr. Van Antwerp: This is a difficult case. It is almost impossible to get the parts thoroughly healed, and when they are healed there is a liability of the pressure causing soreness, and the sore surface will manifest the vicarious action, which is exhibited almost always in this disease. It is a question whether she would wear the appliance after it was fitted on account of this difficulty. The old fashioned springs must be resorted to.

Dr. Cravens described the appliance which he has constructed for this case. It consists of a spring which throws forward two flanges in such a manner as to hook over the edges of the exposed bone. (The design was altogether unique and although very simple, can not easily be described without a diagram.—Reporter.)

Dr. McKellops: Dr. Kingsly ought to be entrusted with all such cases as that. It is very hard to teach the patient to use such an appliance; it takes months to learn to talk with it.

Dr, Smith spoke of the value of springs in such cases. He thought their use was often unavoidable, and they frequently perform a valuable service. He spoke of a case in which he had tried in vain to support the plate by the ordinary methods. He finally succeeded, to his own and the patient's satisfaction, by using springs. There are, no doubt, a great many plates being worn with great discomfort which might have been comfortably supported with springs. In mechanical dentistry, as in operative dentistry, there is a very laudable disposition on the part of intelligent practitioners to retrace their steps and avail themselves of old-fashioned methods, which we can not afford to discard.

Dr. Taylor: I agree with Dr. Smith. Any man who practices dentistry is sure to meet with difficult cases of that kind. I know that there are cases where springs are advantageous. However, I have not used them for twenty years.

Dr. Wheeler, speaking of the merits of rubber and platina for lower sets, said: There are cases where platina is better than rubber, because you can get a better fit. I use very little gold for plates. I think a very good set can be made on gold with rubber attachment. I give the preference to platina always, excepting sometimes for partial sets, when I prefer other materials.

Dr. Smith: In relation to springs, I knew a gentleman who had a partial lower set of teeth which it was impossible for him to wear, because he could not keep them in place. They caused nausea for months; the moment the set was placed in his mouth he experienced a sensation as if he were going to vomit. Springs were attached and he wore them without further inconvenience. For lower plates platina is often better than rubber.

Dr. Taylor: I would like to know whether time has determined the fact that air chambers are an absolute necessity. I have seen mouths much injured by their use. Have had cases where the air chamber was a deteriment to their use, and have found many cases where I got along better without them.

Dr. Baxter: I have often had to cut down the air chamber and attach springs instead.

Dr. Wheeler: Had a lady patient who broke a spring of her plate; I took off the remaining spring, and she has since worn them without any springs at all. I do not think it would have been possible for her to support the plate without springs in the first place.

Dr. Berry: The first full set of teeth I ever made—which was for a patient in whose mouth the alveolar border was completely absorbed away—I supported with springs. I had previously failed with an atmospheric plate. She wore the plate two years or more when one spring broke, and she has never had it replaced.

Dr. Smith: Some ten or twelve years ago, I remember Dr. Goddard told how he used to overcome these difficulties. He said that when a plate would not remain in position, he tied up his patient's jaws with a handkerchief, and told him to go away for a few days, and repeated this method each time he came complaining. (Laughter.) There are many little points concerning the practice of mechanical dentistry, which are worthy of practical consideration. I query whether it is always best to take impressions in plaster. I know a dentist in this city who succeeds admirably in getting fits from wax impressions. It is a question whether wax impressions are not better for lower sets where the ridge is not well marked. Ought the muscles to be pressed out of the way in taking impressions?

Dr. Goddard spoke of a case of a lady who had great difficulty in sustaining her plates. He instructed her to practice reading before a mirror. She did so and finally succeeded in supporting the plates comfortably.

### AFTERNOON SESSION.

Dr. W. N. Morrison read a paper on replanting extracted teeth, which elicited considerable discussion.

Dr, Rawls spoke at considerable length, taking the position that the replanting of extracted teeth, if long deferred, would not be attended with successful results. If the tooth stands irregular in the arch, the socket may be drilled out so that the tooth may be reinserted in a more desirable position.

Dr. Watt referred to Dr. Riggs' treatment for the removal of calculus, an operation which sometimes involves the scraping of the root to its apex, and consequently the removal of the periosteum. From this he infers that a tooth may be sustained in its socket even though the periosteum may to a considerable extent have been removed.

Dr. J. Taft: The greatest interest that attaches to this subject does not perhaps lie in the fact that a tooth that has been extracted will become firmly attached in its socket after being replaced, but in the great lesson we may learn of the recuperating and restoring power of nature.

We are all too much inclined to overlook the functions and possibilities of nature as manifested in our bodies.

If, as a profession, we can become instructed and stimulated to a proper understanding and appreciation of this and kindred subjects, it will be an occasion for great gratification; because it extends our sphere of usefulness, not only for the present, but gives promise for the future.

When we consider a few facts, it is not surprising that a tooth, the investments of which are comparatively healthy, upon being extracted and replaced should become firmly attached in its socket. The teeth from the deposition of salivary calculus, or from some diseased condition of the gums and process, become loosened, and are under such circumstances largely detached, and yet by the removal of the cause or causes of such condition, and proper treatment, reparation of the affected parts not only takes place, but a reunion of the tooth takes place, now that its normal firmness is attained. This is far more difficult of attainment between parts that have been much diseased, than between those in a state of health. Parts are often, by the surgeon, and frequently by accident, separated, and then brought together, readily unite as perfectly as before separation.

The retention of the periosteum on the roots of extracted teeth is not necessary for their insertion and perfect attachment; and hence in those cases in which the teeth are involved in alveolar abscess or other disease of the periosteum, it is quite practicable to remove the diseased tissue even from the entire surface of the root, when upon replacement the tooth will become firmly fixed in place.

Dr. Riggs' treatment of diseased gums has a bearing on this subject, and may be profitably studied in that direction.

Dr. McCollum: Many things have happened during my lifetime which have upset all my theories. Iron has been made to swim. Nothing seems to be impossible, so I am prepared to believe almost any statement in regard to the restorative powers of nature. As to drilling out the cavity to change the direction of the socket before replanting the root, I should be afraid of injuring the tissue; the operation must certainly be painful. I have had but little experience in replanting teeth. I once extracted a sound tooth by accident, for a miss aged fourteen, replaced it, and it has remained in position for ten years; it was replaced in two minutes after extraction.

Dr. Rawls: I do not doubt that, as Dr. Taft says, the periosteum can be restored in some cases, but they are very few and far between. In most cases where the periosteum becomes involved the teeth will finally drop out. This disease and susceptibility runs through whole families.

Dr. McKellops: Porcelain teeth have been planted in the socket of extracted teeth, but with no success. But as regards the replanting natural teeth I believe it can be done with perfect success. It will be in the same condition as a tooth from which you have removed the nerve and filled the root. I do believe that a natural tooth may be replaced and grow in position, after being out of the socket a loug time.

Dr. Taft: When a tooth is deprived of its pulp, and its supply from within is taken away, (and this sometimes occurs even when the outer connection is unbroken) there will be a change in one or more of its constituents. This accounts for the change of color which is observed. This change of color is more noticeable in the teeth of the young. Why does not an extracted tooth always exhibit this peculiar change of color? Because the material in the tooth does not undergo decomposition. It may be said that it is a dead tooth, but are we warranted in that conclusion? Since there has been no decomposition, it is not altogether unreasonable expect to it to unite with its native tissues and renew its life and functions, as the seed does under favorable circumstances. Who shall say this is impossible? So long as there is no change in the constituents, or soft-solid substance of a tooth, there is an ability to unite with the living membranes of the socket.

Dr. McCollum: In tropical countries there are plants which at times dry up and wilt and appear to be dead, but when exposed to moisture, they expand and exhibit an active growth, Are the teeth not endowed with this principle of life?

Dr. Baxter: A tooth, as I understand it, is composed of animal and mineral elements. Can we ascertain the connection between these constituents? Here is something we do not understand as yet. It is like the process of galvanism; we see the film deposited, and we do not know exactly how it is done.

Dr. Meredeth: We have all noticed how friable teeth become after they have been extracted. It is a natural result of a deprivation of nourishment. It seems altogether improbable that such teeth can be replanted and become firm. No one will dispute the possibility of a tooth being replanted while its membranes are alive. I have done it successfully in several instances.

Dr. H. A. Smith: Success in the replanting of teeth depends upon the proper observance of certain physiological laws. If a tooth is replanted with the periosteum or any portion of it, while it yet retains, so to speak, the vital principle, and proper rest and support given to the tooth, union and a grade of vitality may be obtained, and the tooth remain in a condition of comparative health. That the functions of the pulp can be renewed after being servered from its vascular and nervous connections, does not seem quite reasonable. Dr. Taft spoke of the difference observed in the appearance of an extracted tooth and one retained in the mouth in which the pulp was dead, and suggests that no decomposition occurs in the former. Let us examine this statement. A tooth out of the mouth seemingly undergoes no change, and yet the organic constituents do decay, but the change stops short of putrefaction. The animal matter is dried up or oxydised, and there is no metamorphosis or change in these elements, which would cause them to assume a darker hue, as most likely would result in a dead tooth retained in the mouth. Decay always precedes putrefaction, and in the first instance the change stops short of putrefaction. Then again, as soon as extracted, the great bleaching agent, oxygen begins at once to whiten the tooth. If this statement is correct, then very soon the whole vital activity of the extracted tooth will have gone out of it, and there can be no analogy, as mentioned, between the teeth and the seed of the plant. The seed may, under favorable circumstances, remain in a state of absolute inactivity for a long time, but when exposed to air and moisture, with a higher temperature, it quickly germinates. In animal life, the ovum, like the seed of the plant, contains a store of appropriate nutriment, previously elaborated by the parent, which serves for the development of the embryo, up to the period when it can obtain and digest food for itself. Here the analogy in a great measure ceases. There would appear to be more agreement between the dead tooth and the fallen leaf of the plant. Although I do not think that replantation can be as generally practiced as some here would have us believe, yet I would not discourage any one from making the attempt in cases where success seemed at all probable, for it is those who believe that things can be done who do them.

Dr. Morrison: I have seen teeth replanted with apparent success after being out of the mouth forty-eight hours. I have in some cases enlarged the cavity by drilling in preference to cutting off the root. Do not remove all the periosteum.

Dr. Leslie told of a dentist in Boston, who extracted a tooth for himself, filled it, and had it replaced by another dentist. The operation was a success. He had seen failures, which were marked by absorbtion of the gums and process, and necrosis of the roots. Dr. Bing of Paris practices transplanting in some cases from one month to another. I think the union between the ruptured parts in such cases is simply a mechanical union.

Dr. Watt: I would not enlarge the socket to facilitate getting the root back. It is not at all improbable that the broken filaments of nerve should re-unite. Large portions of nerves in other parts of the system have been excised and the interval has after a while been filled up with new nerve tissue.

Dr. Taylor: We know that teeth have grown fast in the sockets after being transplanted and replanted. Dr. Hunter, you remember, performed this operation. It was abandoned because of the danger of transmitting constitutional diseases. The question in my mind is whether the nerve survives the operation. Is a union established through the minute foramen at the apex of the root? Again: if we scrape off the periosteum, how can there be a perfect union of the denuded root with the membranes of the socket?

When we find recession of the gums at the neck of the tooth, and we remove the tartar and scrape off the periosteum, to what extent do we have a reproduction of the gum?

We have sometimes, I think, a reformation of the process. I have had for two years past, a large number of cases of what the French call "*epulis*," at the neck of the tooth. Sometimes the gum is found to be separated from the neck of the tooth and pus exudes; small specks of foreign matter will be found upon the neck. Cleanse these off thoroughly and inject iodine and carbolic acid until a perfect union is induced.

Dr. Goddard cited a case of a young miss, who, at the age of thirteen, had two incisors knocked out. He replaced them *forty-five years ago*, and to-day they are in position and show no discoloration.

Dr. Morrison: The success of the operation of the replanting of teeth is modified by the age of the patient. In one case there will be no sense of soreness after a few hours; in another, the uneasiness and soreness may continue for two or three weeks. Teeth which have been replanted, after being filled, are less liable to ulcerate than other teeth whose roots have been filled in the mouth.

Dr. Taft: The facility with which union would take place depends upon the readiness with which, in any individual, the coagulable lymph is organized. If there is a deteriorated condition of the lymph, or a low state of vitality in the patient, the result will not be so favorable. The state of the health can be read in the eye; the condition of the gums; the general appearance of the countenance, etc. We will find a considerable number of cases, where systemic poisons exist, or the unfavorable general conditions such as preclude the possibility of a successful performance of the operations of this kind.

